# Clinical Characteristics and Surgical Outcomes of Intradural Spinal Tumor Resections: A Retrospective Single-Center Study

**DOI:** 10.3390/jcm15103669

**Published:** 2026-05-10

**Authors:** Anastasija Krzemińska, Sara Chmielewska, Marta Koźba-Gosztyła, Bogdan Czapiga

**Affiliations:** 1 Department of Neurosurgery, Wroclaw 4th Military Clinical Hospital, Weigla 5, 50-981 Wroclaw, Poland; sarachmie@gmail.com (S.C.); martakozba1@o2.pl (M.K.-G.); bogdan.czapiga@pwr.edu.pl (B.C.); 2 Faculty of Medicine, Wroclaw University of Science and Technology, Grunwaldzki Square 11, 51-377 Wrocław, Poland

**Keywords:** spinal cord tumor, neurological complications, surgery timing, intraoperative neuromonitoring

## Abstract

**Objective**. The aim of this study was to evaluate clinical characteristics, surgical outcomes, and factors influencing postoperative neurological status in patients undergoing resection of intradural spinal tumors, with particular emphasis on the role of preoperative neurological function and intraoperative neuromonitoring (IONM). **Methods**. We conducted a retrospective analysis of 108 patients who underwent surgical resection of intradural spinal tumors at a single neurosurgical center. Patients were categorized into intradural extramedullary (IDEM) and intramedullary (IM) tumor groups. The primary endpoint was the occurrence of a new or worsened postoperative motor deficit at discharge. Secondary endpoints included postoperative sphincter dysfunction and functional status assessed using the modified McCormick scale at discharge and at 6-month follow-up. Categorical variables were compared using the Chi-square or Fisher’s exact test, and continuous variables using the Mann–Whitney U test. **Results**. A total of 108 patients were included (61.1% female; mean age 55.7 ± 15.6 years). IDEM tumors accounted for 77 cases, while 31 were intramedullary. There were no significant differences between IDEM and IM tumors in the rate of new or worsened postoperative motor deficits (9.1% vs. 6.7%, *p* = 1.000), postoperative sphincter dysfunction (2.6% vs. 0%, *p* = 1.000), or functional outcomes assessed using the modified McCormick scale at discharge (*p* = 0.85) and at 6 months (*p* = 0.24). Preoperative motor deficit was strongly associated with postoperative motor dysfunction in the overall cohort (86% vs. 14%, *p* < 0.001), with an even stronger effect observed in the IM subgroup (90.9% vs. 9.1%, *p* < 0.001). IONM was used in 34.3% of cases and was significantly associated with tumor location, histopathology, and surgical complexity. However, IONM use was not associated with postoperative motor outcomes (*p* = 0.645). **Conclusions**. Postoperative neurological outcomes following intradural spinal tumor resection are comparable between intramedullary and extramedullary lesions. Preoperative motor deficit is the strongest predictor of postoperative neurological status, particularly in intramedullary tumors, underscoring the importance of early surgical intervention. IONM is preferentially used in higher-risk cases and should be interpreted as a marker of surgical complexity rather than an independent determinant of outcomes.

## 1. Introduction

Approximately one in six primary central nervous system tumors is located in the spinal canal. Based on the affected compartment, 55% of spinal lesions are extradural, 40% are intradural extramedullary, and 5% are intradural intramedullary spinal cord tumors [1]. Depending on the tumor’s location and size, a lesion may cause spinal canal stenosis, leading to compression of the spinal cord and/or spinal roots. This can result in symptoms such as motor and sensory deficits, sphincter dysfunction, and pain. In many cases, the neurological symptoms precede the diagnosis by several months, highlighting the insidious nature of spinal tumor progression.

Despite the significant risk of worsening neurological deficits associated with spinal tumor resection, surgical resection remains the cornerstone of treatment for most spinal tumors, particularly when radiological or clinical signs of spinal cord compression are present. Advances in microsurgical techniques, intraoperative imaging, and neurophysiological monitoring have improved the safety of tumor removal; however, the risk of postoperative neurological deterioration—especially in intramedullary lesions—remains significant.

In recent years, intraoperative neuromonitoring (IONM) has gained popularity as a supportive tool in complex spinal surgeries, particularly those involving intramedullary tumors or midline myelotomy [2,3,4,5,6,7]. While its utility in preventing irreversible injury to neural structures is widely recognized, the indications for IONM use and its interpretation remain variable across centers. Furthermore, many real-world decisions regarding IONM implementation are driven by perceived surgical risk and institutional protocols rather than by standardized criteria or randomized evidence [2,8,9,10].

Although predictors of neurological outcome in spinal tumor surgery have been widely described, the interpretation of IONM in retrospective studies remains controversial. In particular, it is unclear whether observed associations between IONM use and outcomes reflect its true clinical impact or rather selection bias related to surgical complexity.

There is a growing need for high-quality, center-specific data describing the clinical characteristics, surgical strategies, and outcomes of patients undergoing spinal tumor surgery. Such data may assist in benchmarking practices, understanding real-world variations, and optimizing patient care.

In this retrospective study, we present our institution’s seven-year experience in the surgical management of intradural spinal tumors. We aim to describe patient characteristics, common tumor types, surgical approaches, and postoperative neurological outcomes—providing insight into clinical patterns, challenges, and decision-making in a tertiary neurosurgical center. We also aimed to better characterize the role and interpretation of IONM in routine clinical practice.

## 2. Materials and Methods

This is a retrospective study, for which no additional patient interventions were performed; therefore, no informed consent from participants was required.

### 2.1. Data Collection

Clinical and neurophysiological medical records of patients undergoing spine surgery at Neurosurgery Department of the 4th Military Hospital in Wroclaw, from January 2018 to December 2024, were reviewed. Patients undergoing surgery for the resection of intradural spinal tumors were included. The exclusion criteria were: (1) patients undergoing only a biopsy of the lesion instead of resection; (2) patients with non-tumorous lesions (such as synovial cysts, arachnoid cysts, abscesses, hematomas); (3) patients with lesions of the craniocervical junction with extension to the spinal canal for which a posterior fossa craniotomy was required for their removal; and (4) patients with extradural lesions.

Two independent researchers thoroughly analyzed the medical data of the patients included in the study, starting from the medical history and physical examination upon admission, through the analysis of surgical procedure descriptions, and concluding with the postoperative follow-up examinations. The following data were retrieved: basic patient characteristics, histopathological type of tumor, tumor location (level and relationship to the dura mater and spinal cord), duration of surgery, approach to the tumor, extent of tumor resection, use of neuromonitoring, and the patient’s neurological status as assessed using a modified McCormick Scale (preoperative, postoperative before discharge from the Neurosurgery Unit, and at 6-month follow-up). The extent of surgical resection was based on the neurosurgeon’s evaluation included in the surgery report and, if available, postoperative magnetic resonance imaging (MRI) findings.

All retrieved data were anonymized before further analysis.

### 2.2. Outcome Measures

The primary endpoint of the study was the occurrence of a new or worsened postoperative motor deficit assessed at the time of discharge (early postoperative period). Motor deterioration was defined as the development of a new motor deficit or the progression of a pre-existing motor deficit compared to the preoperative neurological status.

Secondary endpoints included neurological status at 6-month follow-up, evaluated using both motor function assessment and the modified McCormick Scale. Changes in the modified McCormick Scale were analyzed longitudinally, comparing preoperative status, postoperative status at discharge, and 6-month follow-up.

Transient neurological deficits were defined as new or worsened motor deficits observed in the early postoperative period that resolved by the 6-month follow-up.

Early postoperative deficits were assessed at discharge, while longer-term outcomes were evaluated at 6 months. However, the study design does not allow for a detailed distinction between transient and persistent deficits in a statistical model.

### 2.3. Intraoperative Neuromonitoring

The decision to use IONM was made at the discretion of the operating surgeon and was not based on a standardized institutional protocol. Factors influencing this decision included tumor location, suspected intramedullary involvement, anticipated surgical complexity, and surgeon preference.

IONM was performed using transcranial MEP (tcMEP) and SSEP, along with continuous free-running electromyography (EMG) monitoring, in accordance with established neurophysiological protocols. Neuromonitoring data were interpreted in real-time by a clinical neurophysiologist present in the operating room. A decrease of >50% in MEP amplitude or significant latency changes in SSEPs were considered alarming and were immediately communicated to the neurosurgeon and anesthesiologist. Upon notification of signal deterioration, the neurosurgeon would temporarily halt tumor resection and reassess the surgical field. This could include modifying the trajectory of dissection, minimizing traction, or pausing dissection to allow signal recovery. The anesthesiologist, in turn, would evaluate systemic parameters such as mean arterial pressure, oxygenation, and temperature, to rule out reversible physiological causes of signal changes.

This multidisciplinary response strategy aimed to ensure prompt and coordinated intraoperative adjustments to prevent irreversible neurological damage.

The anesthesiologist avoided the use of anesthetic agents that could influence electrophysiological signals.

Detailed intraoperative neuromonitoring signal data were not consistently available and therefore could not be analyzed.

### 2.4. Statistical Analysis

All analyses were performed using Statistica (TIBCO Software, version 13.3, StatSoft (Europe) GmbH, Hamburg, Germany).

Categorical variables were presented as counts and percentages and compared using the Pearson Chi-square test or Fisher’s exact test, as appropriate, depending on expected cell frequencies. Continuous variables were expressed as mean ± standard deviation (SD) and compared using the Mann–Whitney U test due to non-normal distribution of data.

A univariate logistic regression analysis was performed to evaluate the association between preoperative motor deficit and postoperative motor outcome. Due to the limited number of outcome events, multivariable analysis was not performed to avoid model overfitting.

A *p*-value of <0.05 was considered statistically significant.

## 3. Results

### 3.1. Patient Characteristics

A total of 108 patients who underwent surgical resection of intradural spinal tumors were included in the analysis. The cohort consisted of 66 females (61.1%) and 42 males (38.9%). The mean age at the time of surgery was 55.7 ± 15.6 years.

### 3.2. Tumor Characteristics

The distribution of tumor histopathology and spinal location is presented in Table 1.

Among intradural extramedullary tumors (IDEM) (*n* = 77), the most common histopathological types were meningiomas (*n* = 39) and schwannomas (*n* = 26), accounting for the majority of cases in this subgroup. In contrast, intramedullary tumors (IM) (*n* = 31) were more heterogeneous, with ependymomas (*n* = 7), spinal ependymoma not otherwise specified (*n* = 5), and metastases (*n* = 4) being the most frequently observed entities. Diffuse midline glioma (H3 K27M-mutant) and other rare tumor types were identified exclusively in the intramedullary compartment.

**Figure 1:**
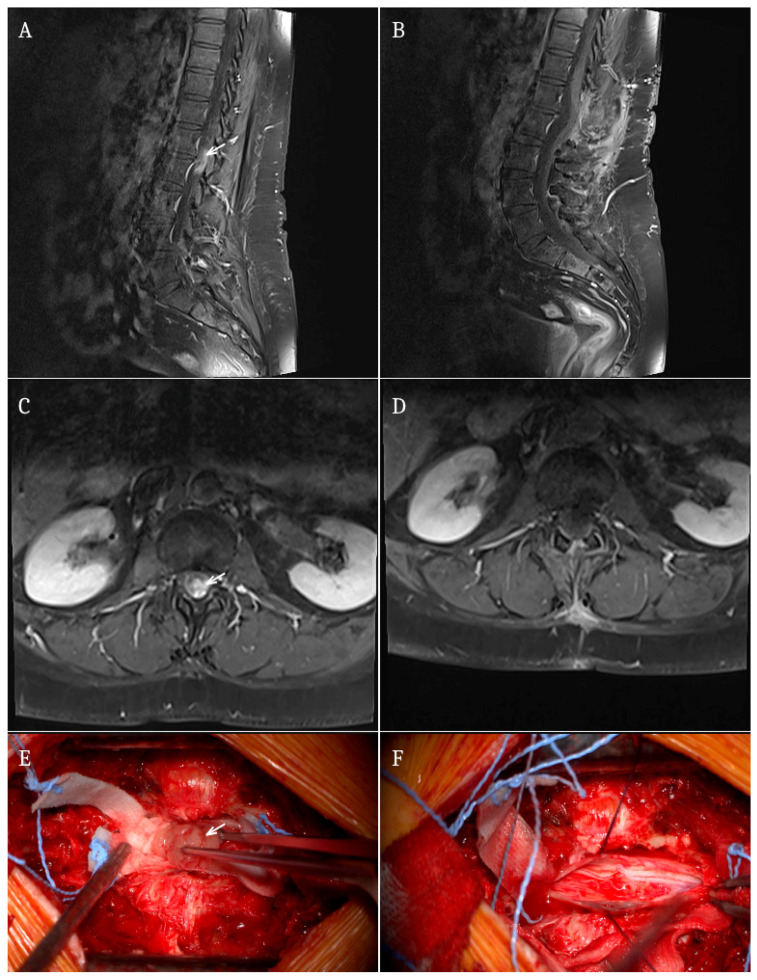
Intradural extramedullary myxopapillary ependymoma at the L1–L2 level: preoperative imaging and intraoperative view. Sagittal (**A**,**B**) and axial (**C**,**D**) contrast-enhanced pre- and postoperative MRI demonstrating an intradural extramedullary lesion at the L1–L2 level (arrows), incidentally detected in a 57-year-old female patient. The tumor appears as a well-defined, enhancing mass causing compression of the cauda equina. Intraoperative photographs (**E**,**F**) show microsurgical exposure and resection of the tumor following durotomy. The lesion was identified within the intradural space and removed using standard microsurgical technique. Histopathological examination confirmed myxopapillary ependymoma. The patient presented without preoperative motor deficits and remained neurologically intact after surgery. Transient postoperative sphincter dysfunction (urinary retention) was observed, which resolved completely within several weeks.

Myxopapillary ependymomas were observed in both IDEM (*n* = 5) and IM (*n* = 2) locations. Several rare tumor types, including ganglioglioma, pilocytic astrocytoma, germinoma, and haemangioblastoma, were found only in the intramedullary group. Figure 1 illustrates a representative case of a patient with myxopapillary ependymoma from our cohort.

### 3.3. Preoperative Symptoms

Preoperative neurological symptoms are summarized in Table 1. Motor deficits were present in 35 patients (45%) with IDEM tumors and 12 patients (40%) with IM tumors. Sensory deficits were observed in 22% and 27% of patients, respectively, while paresthesia was reported in 32% of IDEM and 37% of IM cases.

Back pain was a common presenting symptom in both groups, affecting 45% of patients with IDEM tumors and 50% of those with IM lesions. Sphincter dysfunction was more frequently observed in patients with IM tumors (17%) compared to those with IDEM tumors (5%).

### 3.4. Postoperative Motor Outcomes

New or worsened postoperative motor deficits occurred in 7 patients (9.1%) in the IDEM group and in 2 patients (6.7%) in the IM group. There was no statistically significant difference in the rate of postoperative motor deterioration between these two groups (Fisher’s exact test, *p* = 1.000) (see Table 2).

In a univariate logistic regression analysis, preoperative motor deficit was a strong predictor of postoperative motor dysfunction (OR = 33.3, 95% CI: 11.1–99.5, *p* < 0.001). Moreover, tumor location (intramedullary vs. extramedullary) was not significantly associated with postoperative motor deficit (OR = 0.69, 95% CI: 0.14–3.52, *p* = 0.65).

### 3.5. Postoperative Sphincter Dysfunction

New postoperative sphincter dysfunction was observed in 2 patients (2.6%) in the IDEM group and in none of the patients with IM tumors. There was no statistically significant difference between the groups (Fisher’s exact test, *p* = 1.000).

### 3.6. Postoperative Functional Outcome

Postoperative neurological status, assessed using the modified McCormick scale, did not differ significantly between patients with IDEM and IM tumors (Mann–Whitney U test, *p* = 0.85).

### 3.7. Long-Term Functional Outcome

At 6-month follow-up, there was no statistically significant difference in neurological status between patients with IDEM and IM tumors, as assessed by the modified McCormick scale (Mann–Whitney U test, *p* = 0.24).

### 3.8. Association Between Preoperative and Postoperative Motor Deficits

Within the whole cohort, a strong association was observed between preoperative and postoperative motor deficits. Patients with preoperative motor deficits were significantly more likely to present with motor dysfunction after surgery compared to those without preoperative deficits (86% vs. 14%, *p* < 0.001) (see Table 3).

In the IM subgroup, preoperative motor deficit was strongly associated with postoperative neurological status. Among patients without preoperative deficits, only 9.09% had motor dysfunction postoperatively, compared to 90.9% of patients with preoperative deficits (*p* < 0.001).

### 3.9. Patterns of IONM Use

IONM was used in 37 out of 108 procedures (34.3%). Its use was not random and was significantly associated with specific clinical and tumor-related factors.

IONM was more frequently applied in cervical spine procedures (*p* = 0.002) and in IM tumors (*p* < 0.001), whereas it was less commonly used in lumbosacral lesions (*p* < 0.001) and IDEM tumors (*p* < 0.001).

Table 4 presents patterns of IONM use and should be interpreted as a descriptive analysis rather than a causal model.

No significant association was observed between IONM use and preoperative motor deficit (*p* = 0.413).

The use of IONM also differed significantly according to tumor histopathology (*p* = 0.003), with higher utilization in tumors such as ependymomas and lower use in schwannomas.

Importantly, IONM use was not associated with postoperative motor deficit (*p* = 0.645).

## 4. Discussion

This study presents a comprehensive analysis of clinical characteristics, surgical outcomes, and patterns of IONM use in a cohort of patients undergoing resection of intradural spinal tumors. Several important findings emerge from our data.

First, we found no significant differences in postoperative neurological outcomes between patients with IDEM and IM tumors. The rates of new or worsened postoperative motor deficits, sphincter dysfunction, and functional status assessed by the modified McCormick scale were comparable between the two groups, both at discharge and at 6-month follow-up. These findings suggest that, despite the greater technical complexity traditionally associated with IM tumor surgery, overall functional outcomes may be similar when appropriate surgical strategies are applied [11,12]. The lack of statistically significant differences between intramedullary and extramedullary tumors may reflect limited statistical power, particularly given the relatively small size of the intramedullary subgroup.

The association between preoperative neurological status and postoperative outcome is well established. Patients presenting with preoperative motor deficits had a markedly higher likelihood of persistent postoperative motor dysfunction compared to those without deficits. This relationship was particularly pronounced in the IM subgroup, where the majority of patients with preoperative deficits continued to exhibit postoperative impairment. These findings are consistent with previous reports indicating that preoperative neurological status is the most reliable predictor of postoperative functional recovery. Several authors have reported that patients with preoperative deficits have a significantly lower likelihood of functional recovery, particularly in intramedullary lesions [13,14,15,16,17,18,19]. The strength of this association was further confirmed by logistic regression analysis, demonstrating a markedly increased risk of postoperative motor deficit in patients presenting with preoperative neurological impairment (OR = 33.3). However, due to the limited number of outcome events, multivariable adjustment was not feasible.

Additionally, the heterogeneity of tumor histopathology, particularly within the IM group, may have influenced outcomes independently of anatomical location.

From a clinical perspective, this observation has important implications. It suggests that neurological deficits at presentation reflect underlying spinal cord damage that may be only partially reversible, even after technically successful tumor resection. Consequently, delaying surgical intervention until the development of significant neurological impairment may reduce the potential for functional recovery. Our results therefore support the concept of timely surgical treatment, particularly in patients with lesions at risk of causing progressive neurological deterioration.

No significant difference in postoperative motor outcomes was observed between IM and IDEM tumors. However, the wide confidence interval suggests limited statistical power, particularly due to the small number of intramedullary cases and outcome events.

Another key aspect of this study is the evaluation of IONM use. Although a higher rate of postoperative motor deficits was observed in patients undergoing surgery with IONM, this association was not statistically significant and should not be interpreted as a causal effect. Instead, our analysis clearly demonstrates that the use of IONM was not standardized and largely depended on surgeon preference and perceived surgical complexity. Factors such as intramedullary tumor location, cervical level, and the anticipated need for myelotomy influenced the decision to apply IONM. This explains why neuromonitoring was used inconsistently even within similar tumor types or anatomical locations. Therefore, IONM use in this study should be interpreted as a surrogate marker of surgical difficulty rather than a controlled variable. This pattern reflects confounding by indication, where neuromonitoring is selectively applied in cases with an inherently higher risk of neurological deterioration.

Importantly, no significant association was found between IONM use and postoperative motor outcomes. These findings suggest that IONM should be viewed primarily as a tool supporting intraoperative decision-making rather than as an independent determinant of surgical outcome. Its role may be particularly relevant in complex cases where real-time functional feedback can guide surgical strategy and potentially prevent irreversible injury.

The lack of association between IONM use and postoperative outcomes observed in our study should be interpreted with caution. Similar findings have been reported in previous retrospective studies, where IONM was preferentially used in more complex cases, leading to confounding by indication [20].

Taken together, our results indicate that tumor location (intramedullary vs. extramedullary) and histopathology are less important determinants of outcome than preoperative neurological status. This emphasizes the need for individualized treatment strategies that consider both tumor characteristics and the patient’s baseline neurological function.

### Study Limitations

Several limitations of this study should be acknowledged. First, the retrospective design introduces an inherent risk of selection bias and limits the ability to establish causal relationships. Second, although the overall cohort size is relatively large for a single-center study, subgroup analyses—particularly within the IM group—are based on a limited number of patients and events, which may reduce statistical power.

Third, the use of IONM was not standardized and was left to the discretion of the surgical team, resulting in non-random allocation and potential confounding by indication. Additionally, the lack of IONM data (e.g., signal changes, response to alerts) limits the ability to assess its true clinical effectiveness. Therefore, conclusions regarding the effectiveness of IONM should be interpreted with caution.

Fourth, functional outcomes were primarily assessed using the modified McCormick scale, which, while widely used, may not fully capture subtle changes in neurological function or quality of life.

Fifth, the statistical analysis was primarily univariate. Although this approach allowed for clear descriptive comparisons, it limits the ability to determine independent predictors of postoperative outcomes. A multivariable analysis was not performed due to the relatively low number of outcome events, in order to avoid model overfitting. Consequently, potential confounding factors such as tumor location, histopathology, and baseline neurological status could not be fully adjusted for.

Additionally, the IM tumor subgroup was relatively small and heterogeneous, including various histopathological entities with distinct biological behavior. This heterogeneity may have influenced postoperative outcomes and limited the ability to detect subgroup-specific effects.

Sixth, the follow-up period was limited to 6 months, which may not fully reflect long-term neurological recovery, especially in patients with IM tumors. Moreover, postoperative neurological status was primarily assessed at the time of discharge. Although a 6-month follow-up was included, the study design did not allow for a detailed distinction between transient and permanent neurological deficits using time-dependent or longitudinal analytical methods.

Finally, the study may be underpowered to detect small differences between groups, particularly in analyses comparing IM and IDEM tumors, as reflected by the wide confidence intervals.

## 5. Conclusions

Intradural spinal tumor surgery is associated with generally favorable neurological outcomes, with no significant differences observed between intramedullary and extramedullary lesions.

Preoperative motor deficit emerged as the strongest predictor of postoperative neurological status, particularly in patients with intramedullary tumors. These findings highlight that early surgical intervention may be beneficial in preventing the development of irreversible neurological impairment.

Intraoperative neuromonitoring was selectively used in more complex and higher-risk cases and was not independently associated with postoperative motor outcomes. Its use should therefore be interpreted as a marker of surgical complexity rather than a determinant of outcome.

Overall, our results support a treatment strategy focused on timely intervention and individualized surgical planning to optimize functional outcomes in patients with intradural spinal tumors.

## Figures and Tables

**Table 1:** The distribution of tumor histopathology, spinal location and preoperative symptoms.

	Intradural Extramedullary Tumors, *n*	Intradural Intramedullary Tumors, *n*	Total, *n*
Histopathological diagnosis			
Meningioma	39	1	40
Schwannoma	26	0	26
Metastasis	1	4	5
Ependymoma	1	7	8
Myxopapillary ependymoma	5	2	7
Spinal ependymoma, NOS	0	5	5
Cavernous hemangioma	1	1	2
Haemangiopericytoma	2	1	3
Subependymoma	0	2	2
Diffuse midline glioma, H3 K27M Mutant	0	2	2
Neurofibrolipoma	1	0	1
Neurofibroma	1	0	1
Paraganglioma	0	1	1
Mature teratoma	0	1	1
Pilocytyic astrocytoma	0	1	1
Germinoma	0	1	1
Ganglioglioma	0	1	1
Haemangioblastoma	0	1	1
Spinal level			
Cervical	17	14	31
Thoracic	47	13	60
Lumbosacral	20	8	28
Preoperative symptoms			
Motor deficit	35 (45%)	12 (40%)	
Sensory deficit	17 (22%)	8 (27%)	
Paresthesia	25 (32%)	11 (37%)	
Back pain	35 (45%)	15 (50%)	
Sphincter dysfunction	4 (5%)	5 (17%)	

**Table 2:** Postoperative neurological outcomes in patients with intradural spinal tumors, stratified by tumor location (extramedullary vs. intramedullary).

	Intradural Extramedullary Tumors, *n (%)*	Intradural Intramedullary Tumors, *n (%)*	*p*-Value
Occurrence of a new or worsened postoperative motor deficit assessed at the time of discharge	7 (9.09%)	2 (6.67%)	1.000
New postoperative sphincter dysfunction	2 (2.6%)	0	1.000
Postoperative modified McCormick scale score	1.95 ± 1.12	2 ± 1.17	0.85
Modified McCormick scale score at 6-month follow-up	1.53 ± 0.83	1.83 ± 1.1	0.24

**Table 3:** Association between preoperative motor deficits and postoperative motor outcomes in patients with intradural spinal tumors and in the intramedullary subgroup.

Intradural Extramedullary Tumors and Intramedullary Tumors			
	Postoperative Motor Deficit, *n* (%)	No Postoperative Motor Deficit, *n* (%)	*p*-Value
No preoperative motor deficit	6 (13.95%)	54 (84.38%)	<0.001
Preoperative motor deficit	37 (86.05%)	10 (15.62%)	
Total	43	64	
Intramedullary Tumors			
	Postoperative motor deficit, *n* (%)	No postoperative motor deficit, *n* (%)	*p*-value
No preoperative motor deficit	1 (9.09%)	17 (89.47%)	<0.001
Preoperative motor deficit	10 (90.91%)	2 (10.53%)	
Total	11	19	

Values are presented as number (percentage within columns within subgroup). *p*-values were calculated using the Chi-square or Fisher’s exact test, as appropriate.

**Table 4:** Distribution of intraoperative neuromonitoring use according to clinical and tumor characteristics.

	IONM Group, *n*	Non-IONM Group, *n*	*p*-Value
Cervical spine	18	13	0.002
Thoracic spine	21	39	1.000
Lumbosacral spine	2	26	<0.001
Intramedullary tumors	20	11	<0.001
Intradural extramedullary tumors	17	60	<0.001
Preoperative motor deficit	19	29	0.413
Postoperative motor deficit	19	24	0.645
Schwannoma	3	23	0.003
Ependymoma, Subependymoma, Myxopapillary ependymoma, Spinal ependymoma	11	11	
Meningioma	12	28	

IONM—intraoperative neuromonitoring.

## Data Availability

The data supporting the findings of this study are available from the corresponding author upon reasonable request.

## Abbreviations

The following abbreviations are used in this manuscript:

IONM	Intraoperative neuromonitoring
IDEM	Intradural extramedullary
IM	Intramedullary
MRI	Magnetic resonance imaging
MEP	Motor evoked potential
SSEP	Somatosensory evoked potential
